# Supplementary material for: Replication Fork-Associated Checkpoint Mediator Mrc1/Claspin Acts in Arrest-Independent Double-Strand Break Survival
Source: bioRxiv. 2026 Sep 17:2026.09.15.751834. Preprint. [Version 1] doi: 10.64898/2026.09.15.751834 (PMC13596372; doi:10.64898/2026.09.15.751834)
Supplement: Supplementary file 1 [file NIHPP2026.09.15.751834v1-supplement-3.pdf]

bioRxiv preprint doi: https://doi.org/10.64898/2026.09.15.751834; this version posted September 17, 2026. The copyright holder for this preprint (which was not certified by peer review) is the author/funder, who has granted bioRxiv a license to display the preprint in perpetuity. It is made available under a CC-BY 4.0 International license.

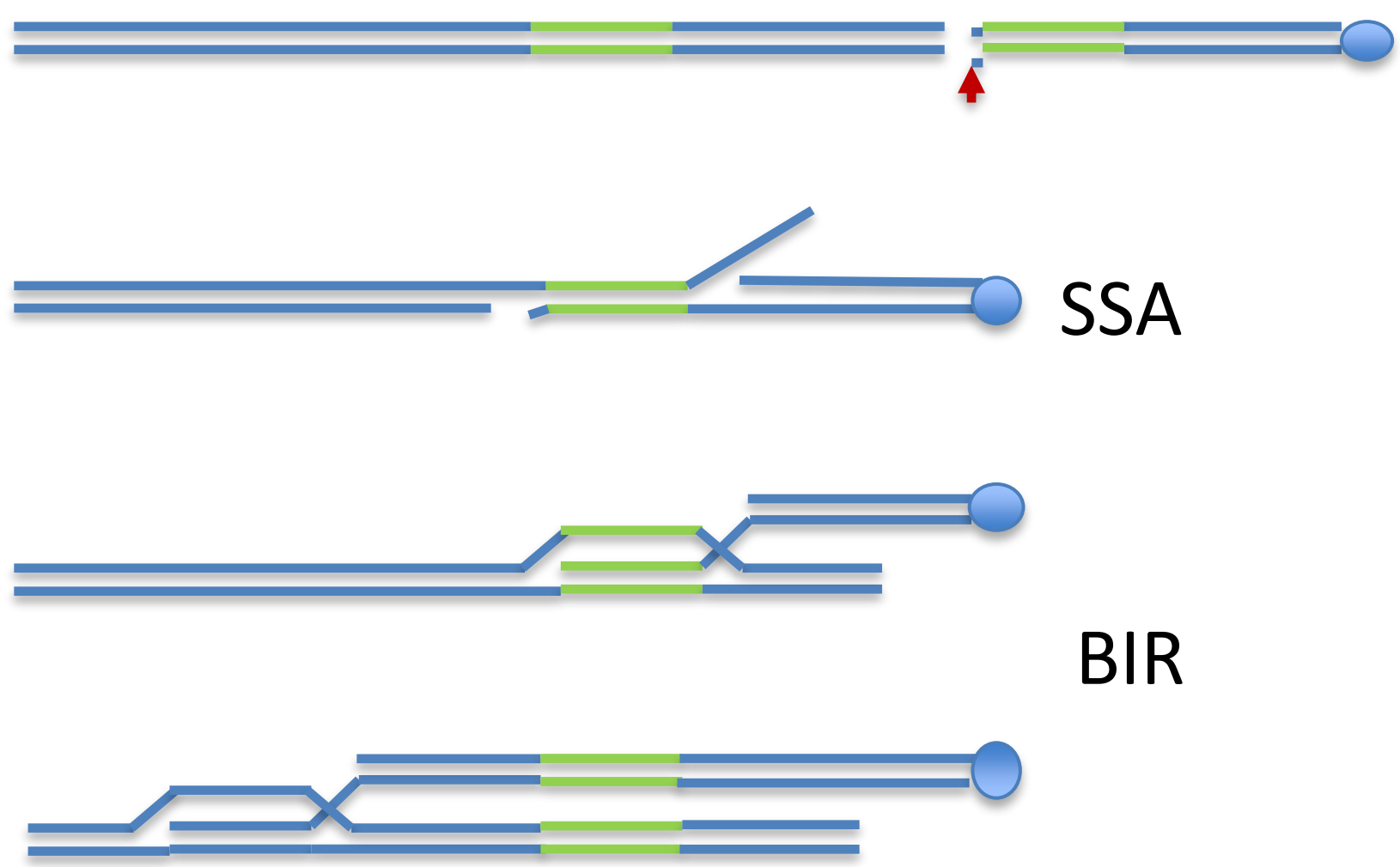

Figure S1 | Alternative repair of a DSB in strain tNS2333. tNS233 can be repaired through BIR or SSA. Although both products are the same, BIR will harbor a large, acentric chromosome fragment until it is degraded.

bioRxiv preprint doi: https://doi.org/10.64898/2026.09.15.751834; this version posted September 17, 2026. The copyright holder for this preprint (which was not certified by peer review) is the author/funder, who has granted bioRxiv a license to display the preprint in perpetuity. It is made available under a CC-BY 4.0 International license.

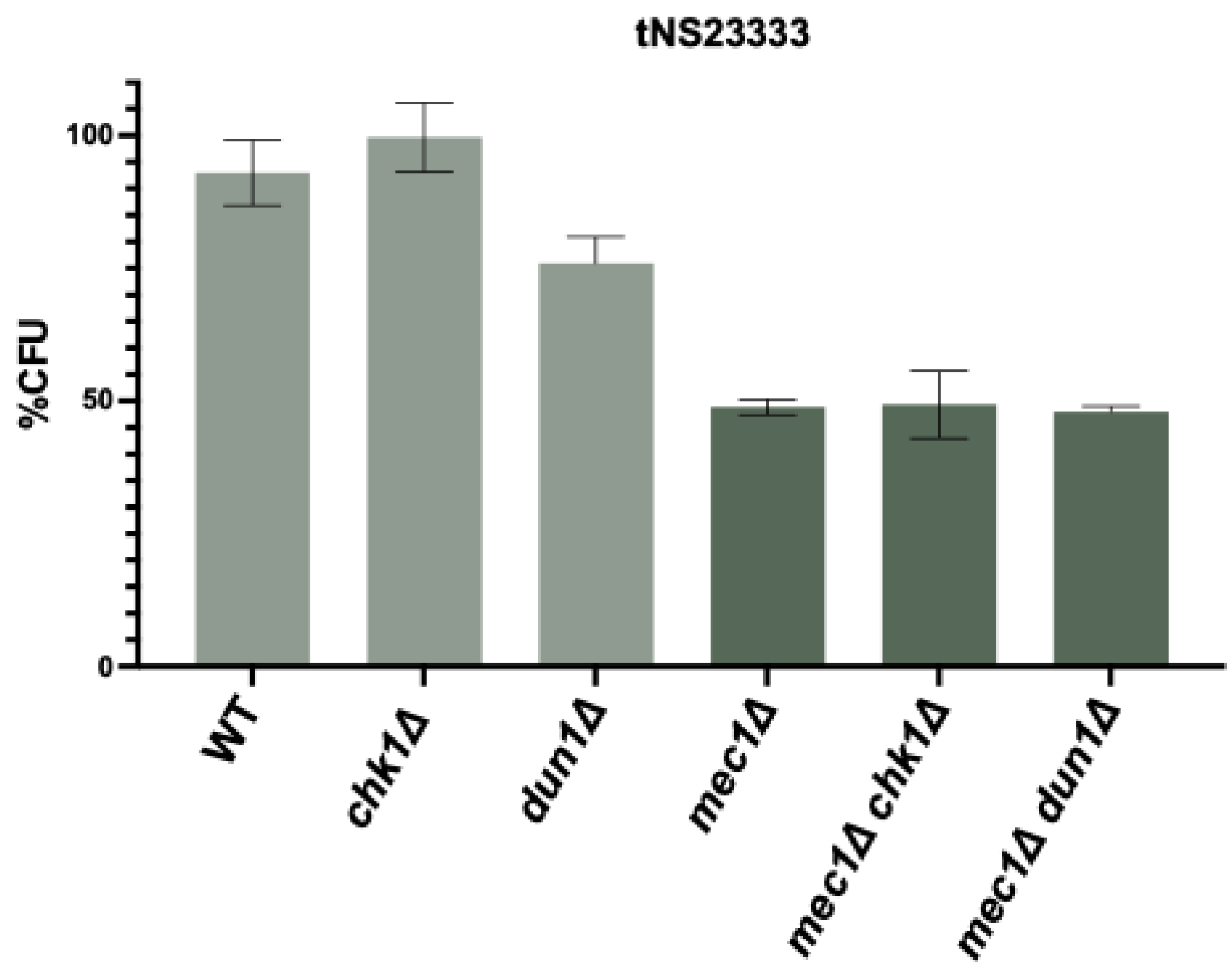

Figure S2 | Neither Dun1 nor Chk1 are important for Mec1-independent survival. CFU assay in tNS233, Chk1 and Dun1 have no impact on Mec1-dependent or independent survival.

bioRxiv preprint doi: https://doi.org/10.64898/2026.09.15.751834; this version posted September 17, 2026. The copyright holder for this preprint (which was not certified by peer review) is the author/funder, who has granted bioRxiv a license to display the preprint in perpetuity. It is made available under a CC-BY 4.0 International license.

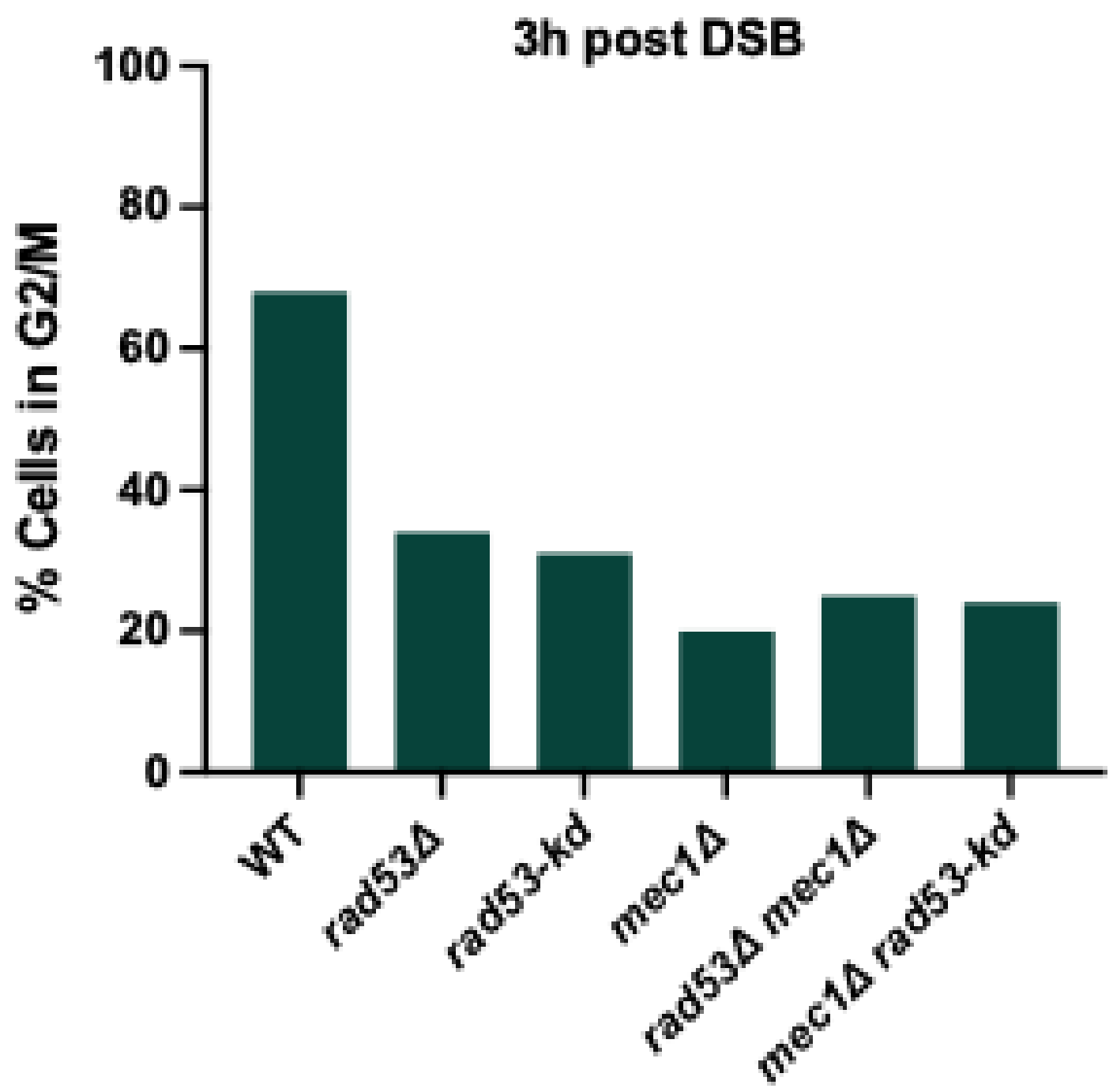

Figure S3 | Rad53-kd mutants do not arrest. Percentage of cells in G2/M arrest 3h after DSB induction in tNS2333. <100 cells collected for each mutant.

bioRxiv preprint doi: https://doi.org/10.64898/2026.09.15.751834; this version posted September 17, 2026. The copyright holder for this preprint (which was not certified by peer review) is the author/funder, who has granted bioRxiv a license to display the preprint in perpetuity. It is made available under a CC-BY 4.0 International license.

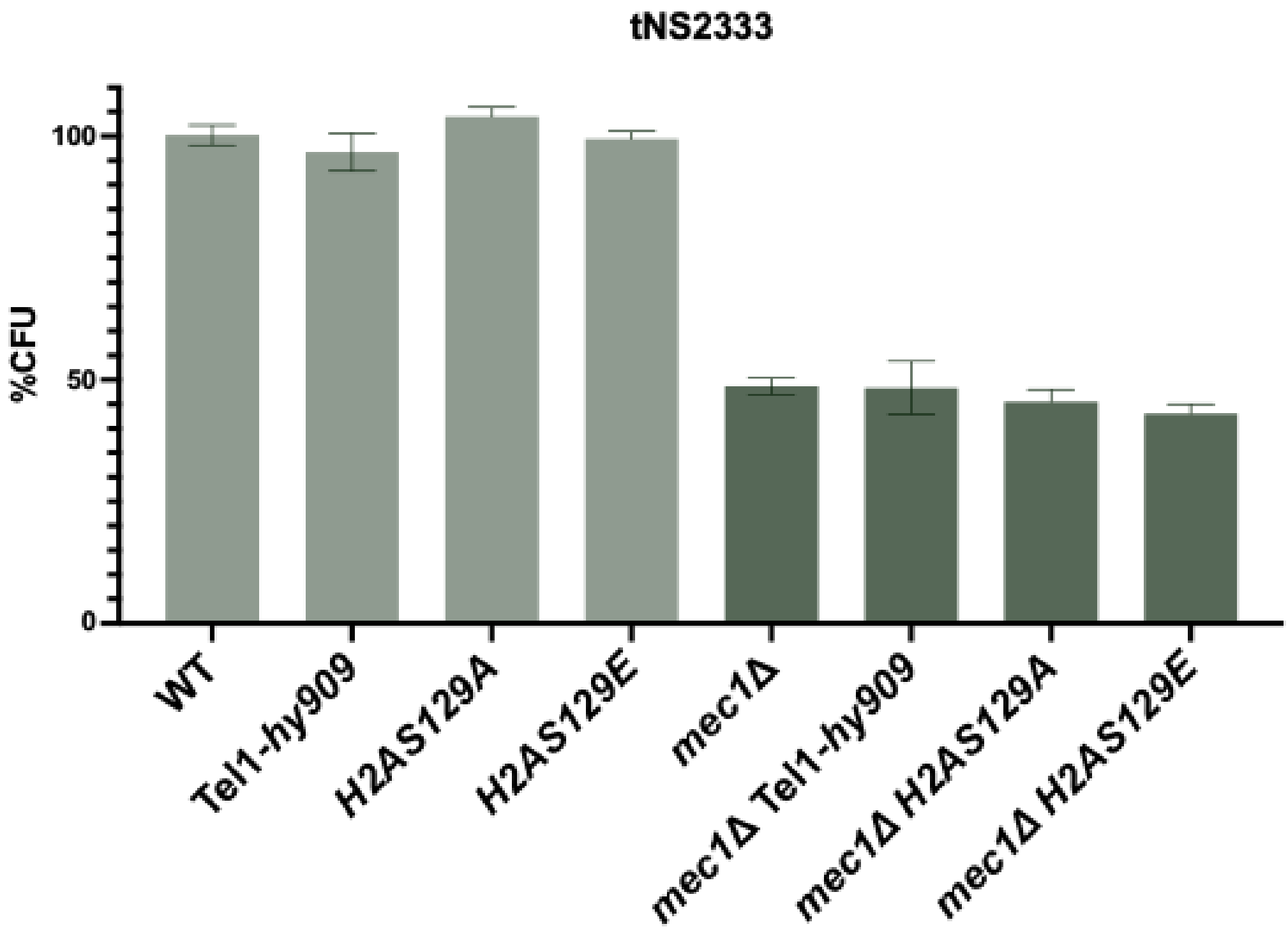

Figure S4 | Neither hyperactive Tel1 nor γ-H2A is important for Mec1-independent survival. CFU assay in tNS2333. hyperactive Tel1 mutant tel1-hy909 shows no improvement for Mec1-independent survival. Neither H2A-S129A nor H2A-S129E impact Mec1-independent survival.

bioRxiv preprint doi: https://doi.org/10.64898/2026.09.15.751834; this version posted September 17, 2026. The copyright holder for this preprint (which was not certified by peer review) is the author/funder, who has granted bioRxiv a license to display the preprint in perpetuity. It is made available under a CC-BY 4.0 International license.

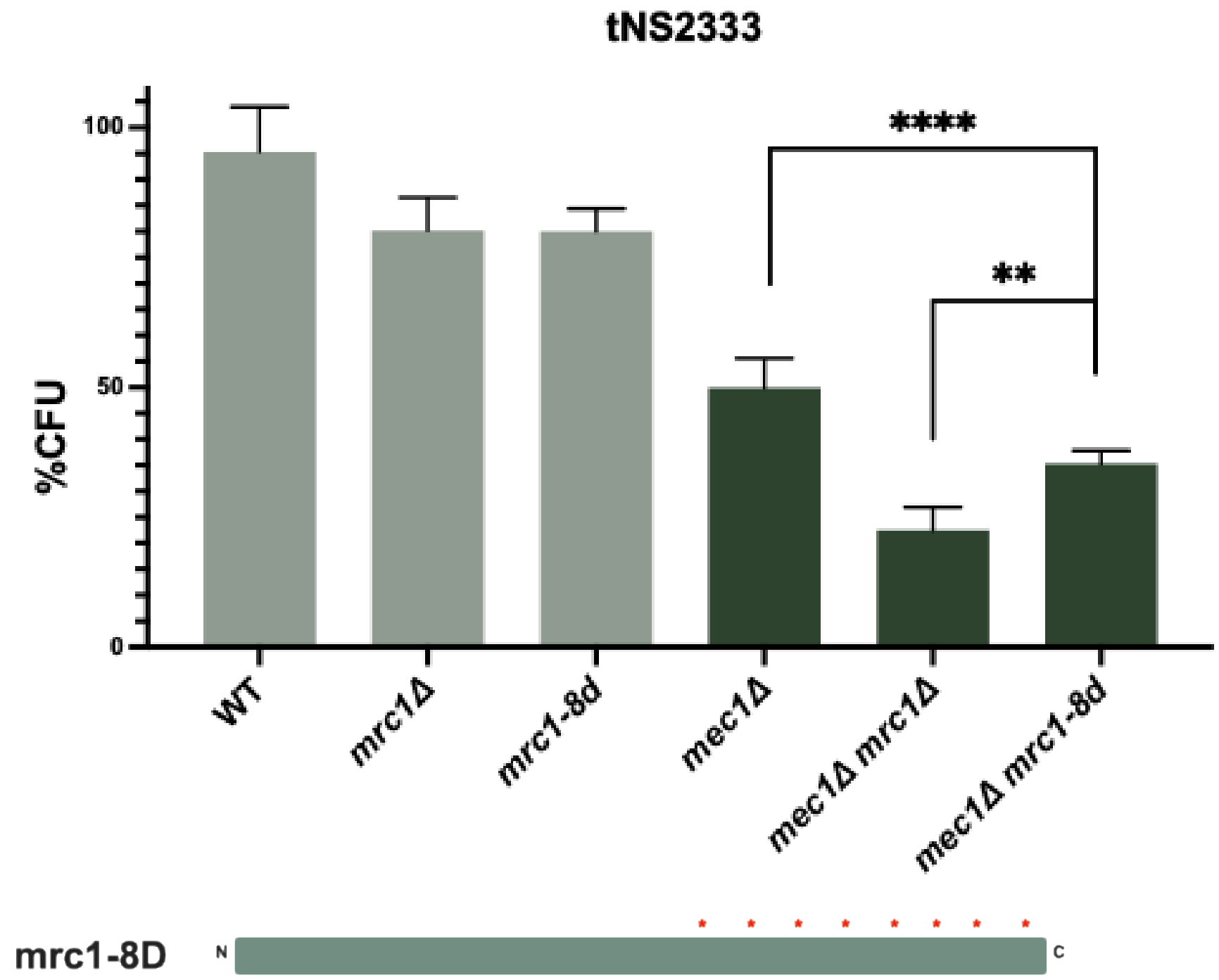

Figure S5 | mrc1-8D shows a partial defect. CFU assay in tNS2333, *mec1*Δ and *mrc1-8D* mutants (8 Rad53 phosphorylation sites→8D).

bioRxiv preprint doi: https://doi.org/10.64898/2026.09.15.751834; this version posted September 17, 2026. The copyright holder for this preprint (which was not certified by peer review) is the author/funder, who has granted bioRxiv a license to display the preprint in perpetuity. It is made available under a CC-BY 4.0 International license.

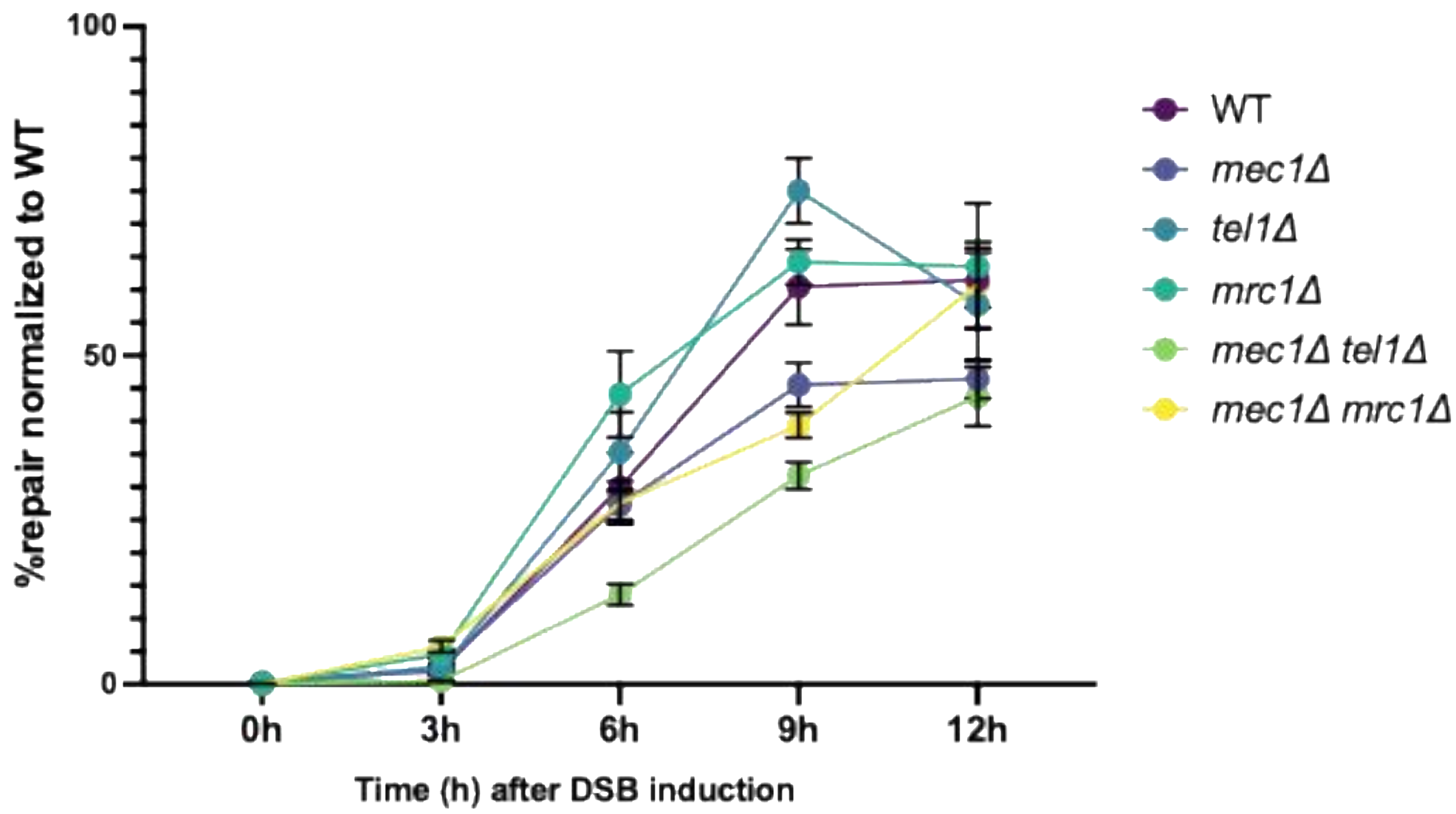

Figure S6 | Nocodazole arrested cells show rescue of repair in *mec1*Δ cells. In tNS2333, *mec1*Δ, *mec1*Δ *mrc1*Δ and *mec1*Δ *tel1*Δ all show rescue of repair in cells arrested in nocodazole 3 h before DSB induction. The y-axis is normalized to the percent PCR signal compared to cells that had previously repaired the DSB (WT colony grown on YEP-GAL) and normalized to a control amplicon at *GLC7.*

bioRxiv preprint doi: https://doi.org/10.64898/2026.09.15.751834; this version posted September 17, 2026. The copyright holder for this preprint (which was not certified by peer review) is the author/funder, who has granted bioRxiv a license to display the preprint in perpetuity. It is made available under a CC-BY 4.0 International license.

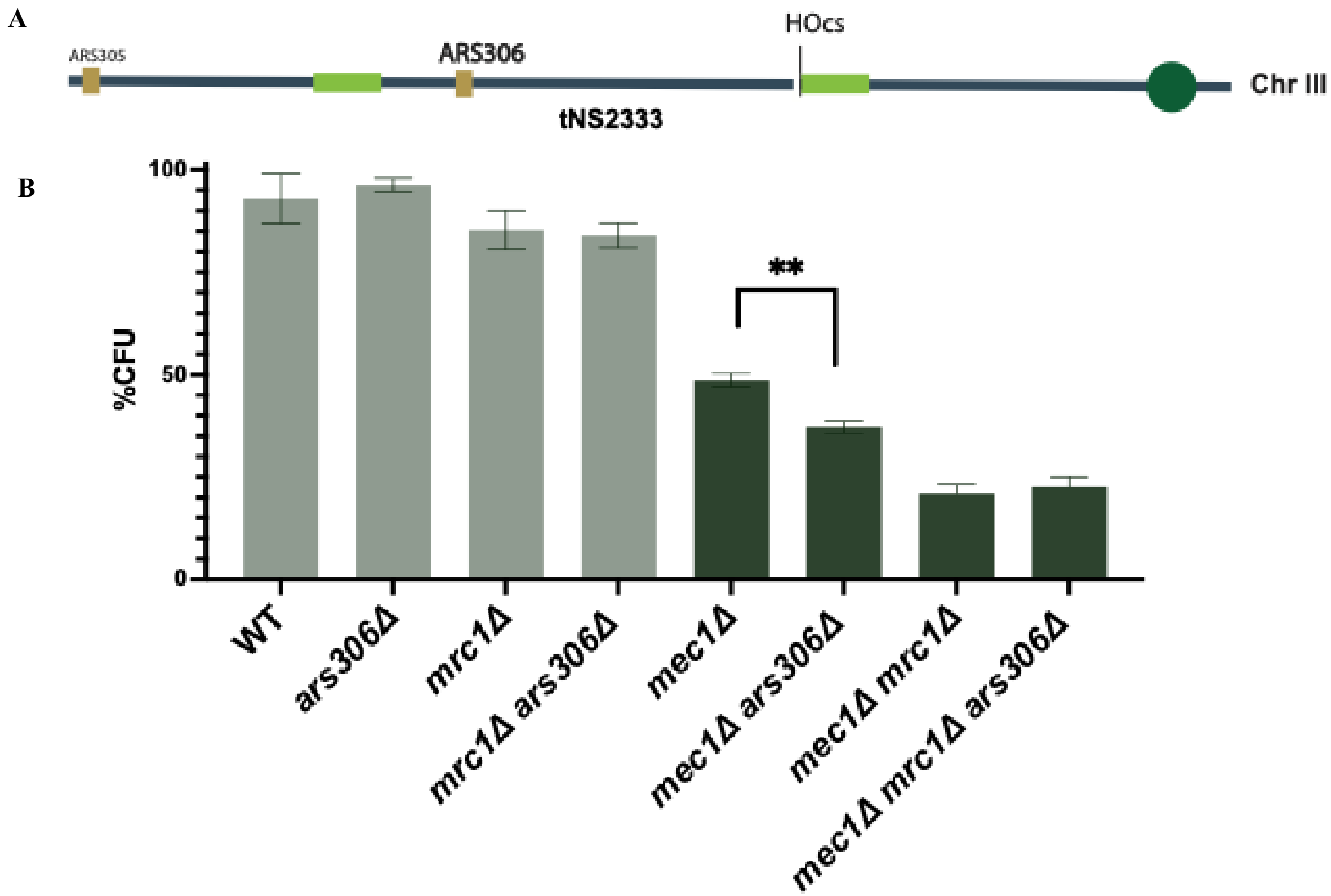

Figure S7 | Loss of replication origin ARS306 significantly reduces survival in *mec1Δ* cells. A. diagram of the location of origins of replication on the left arm of chromosome 3. B. CFU assay in tNS2333.

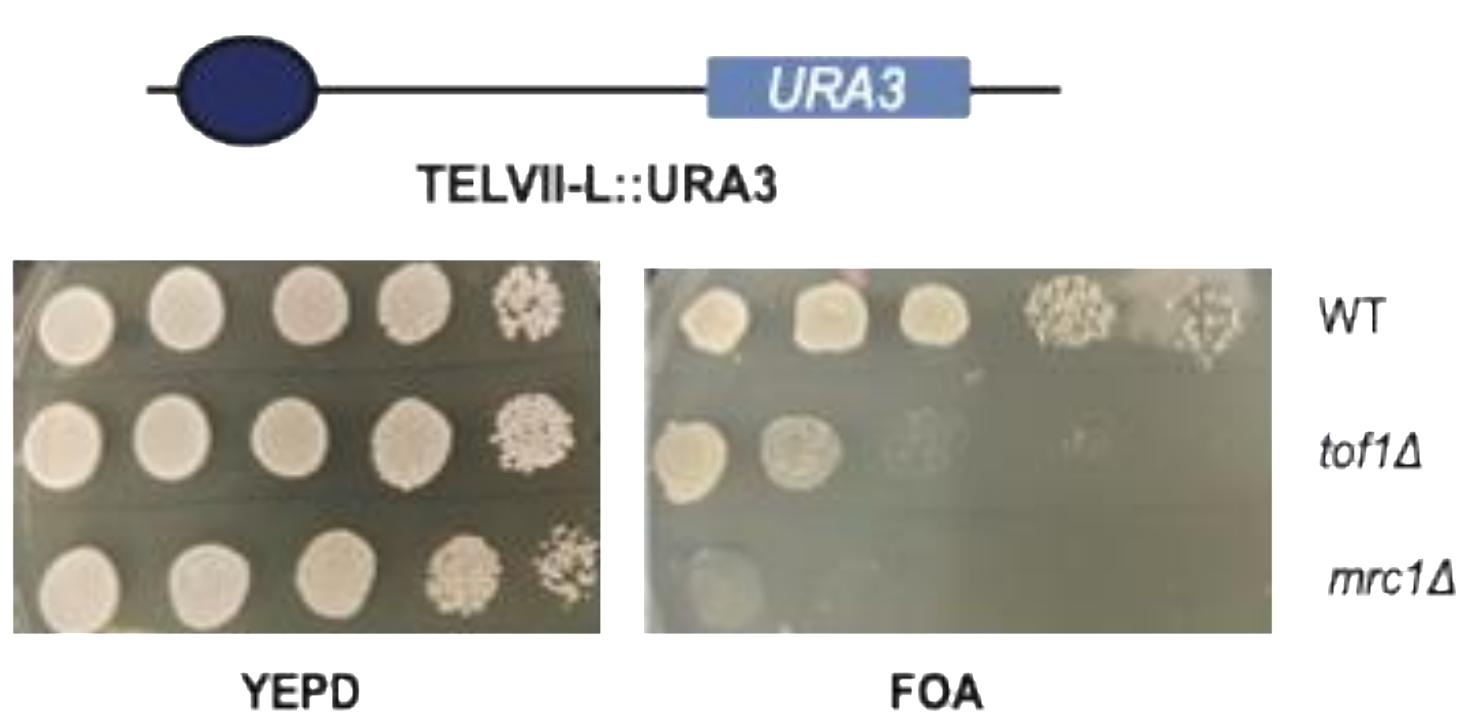

Figure S8 | Loss of silencing at telomeres in *mrc1*Δ is more severe than tof1Δ. A. Telomere silencing assay (adapted from Yu et al., 2024) DMY3314, where a URA3 gene is placed at the telomere. B. Cells plated on FOA vs YEPD. Loss of silencing is measure by ability to grow on FOA plates. *mrc1*Δ leads to significantly more loss of silencing at the telomeres than *tof1*Δ.
