# Supplementary material for: Replication Fork-Associated Checkpoint Mediator Mrc1/Claspin Acts in Arrest-Independent Double-Strand Break Survival
Source: bioRxiv. 2026 Sep 17:2026.09.15.751834. Preprint. [Version 1] doi: 10.64898/2026.09.15.751834 (PMC13596372; doi:10.64898/2026.09.15.751834)
Supplement: Supplementary file 3 [file media-2.pdf]

Supplemental Table 3

Oligonucleotide	sequence	purpose	experiment
Ma315mrc1gRNA2fwd	ACTACCAGATGATTCATAATgtttt	to delete 355-670 aa of mrc1	
Ma316mrc1gRNA2rev	ACTACCAGATGATTCATAATgtttt	to delete 355-670 aa of mrc1	
Ma317mrc1gRNA3fwd	ACTACCAGATGATTCATAATgtttt	to delete 711-850 or 798 aa of mrc1	
Ma318mrc1gRNA3rev	ACTACCAGATGATTCATAATgtttt	to delete 711-850 or 798 aa of mrc1	
Ma319mrc1gRNA4fwd	ACTACCAGATGATTCATAATgtttt	to delete 711-850 or 798 aa of mrc1	
Ma320mrc1gRNA4rev	ACTACCAGATGATTCATAATgtttt	to delete 711-850 or 798 aa of mrc1	
Ma321mrc1gRNA5fwd	ACTACCAGATGATTCATAATgtttt	to delete 869-1096 aa of mrc1	
Ma322mrc1gRNA5rev	ACTACCAGATGATTCATAATgtttt	to delete 869-1096 aa of mrc1	
			Repair PCR
LEU2RP	ACTACCAGATGATTCATAATgtttt	Downstream and antisense to *LEU2*.	Repair PCR
MA193_trpfwdforrepair	ACTACCAGATGATTCATAATgtttt	fwd primer at the end of TRP for repair in tns2333	Repair PCR
EB29GLC7ctrlfwd	ACTACCAGATGATTCATAATgtttt	fwd primer for ctrl	Repair PCR
EB30GLC7ctrlrev	ACTACCAGATGATTCATAATgtttt	rev primer for ctrl	ChIP experiments
MA43ADH1fwd	ACTACCAGATGATTCATAATgtttt	*ADH1* fwd for resection assay ctrl	ChIP experiments
MA44ADH1rev	ACTACCAGATGATTCATAATgtttt	*ADH1* rev for resection assay ctrl	ChIP experiments

MA018MATAlphaFWD	ACTACCAGATGATTCATAATgtttt	*MAT*α FWD primer Immediately distal to HOcs	ChIP experiments
MA19MATalpharevRSA1	ACTACCAGATGATTCATAATgtttt	*MAT*α REV primer Immediately distal to HOcs.	ChIP experiments
MA36_726bpdownstreamofHOcsfwd	ACTACCAGATGATTCATAATgtttt	726bp downstream HOcs fwd	ChIP experiments
MA37_726bpdownstreamofHOcsrev	ACTACCAGATGATTCATAATgtttt	726bp downstream HOcs rev	ChIP experiments
MA38_5_7kbdownstreamofHOcsfw	ACTACCAGATGATTCATAATgtttt	5.7 kb downstream HOcs fwd	ChIP experiments
MA39_5_7kbdownstreamofHOcsrev	ACTACCAGATGATTCATAATgtttt	5.7 kb downstream HOcs rev	
GM335	ACTACCAGATGATTCATAATgtttt	80mer Rad53 K227A	
GM336	ACTACCAGATGATTCATAATgtttt		
GM337	ACTACCAGATGATTCATAATgtttt		
